# The Effectiveness of a Serious Game (MemoreBox) for Cognitive Functioning Among Seniors in Care Facilities: Field Study

**DOI:** 10.2196/33169

**Published:** 2022-04-01

**Authors:** Jana Marina Kleschnitzki, Luzi Beyer, Reinhard Beyer, Inga Großmann

**Affiliations:** 1 Department of Psychology Humboldt-University of Berlin Berlin Germany; 2 Department of Social Work Alice-Salomon-Hochschule Berlin Germany; 3 Department of Business Psychology University of Applied Sciences Berlin Germany

**Keywords:** serious game, cognitive function, mental health, seniors, care facilities, aging, cognitive impairments, health technology

## Abstract

**Background:**

Serious games have been found to have enhancing and preventative effects on cognitive abilities in healthy older adults. Yet, there are few results on the effects in older seniors with age-related low cognitive impairments. Their special needs were considered when designing and using innovate technology in the area of prevention, which is especially relevant owing to the continuously aging population.

**Objective:**

The objective of this study was to evaluate the impact of a serious game on the cognitive abilities of seniors in order to potentially implement innovative resource-oriented technological interventions that can help to meet future challenges.

**Methods:**

In this controlled trial, we tested the serious game MemoreBox, which features modules specifically designed for seniors in nursing homes. Over a period of 1 year, we tested the cognitive abilities of 1000 seniors at 4 time points using the Mini-Mental Status Test. Only half of the participating seniors engaged with the serious game.

**Results:**

The study included an intervention group (n=56) and a control group (did not play; n=55). Based on the in-game data collection, a second intervention group (n=38) was identified within the original intervention group, which exactly followed the planned protocol. There were no noteworthy differences between the demographic and main variables of the overall sample. The large reduction in the sample size was due to the effects of the COVID-19 pandemic (drop-out rate: 88.9%). The CI was set at 5%. Mixed analysis of variance (ANOVA) between the cognitive abilities of the intervention and control groups did not show a statistically significant difference between time and group (*F*_2.710,295.379_=1.942; *P*=.13; partial *η*²=0.018). We noted approximately the same findings for mixed ANOVA between the cognitive abilities of the second intervention and control groups (*F*_3,273_=2.574; *P*=.054; partial *η*²=0.028). However, we did observe clear tendencies and a statistically significant difference between the 2 groups after 9 months of the intervention (t_88.1_=−2.394; *P*=.02).

**Conclusions:**

The results of this study show similarities with the current research situation. Moreover, the data indicate that the intervention can have an effect on the cognitive abilities of seniors, provided that they regularly play the serious game of MemoreBox. The small sample size means that the tendency toward improvement cannot be proven as statistically significant. However, the tendency shown warrants further research. Establishing an effective prevention tool as part of standard care in nursing homes by means of an easy-to-use serious game would be a considerable contribution to the weakened health care system in Germany as it would offer a means of activating senior citizens in partially and fully inpatient care facilities.

**Trial Registration:**

German Clinical Trials Register DRKS00016633; https://tinyurl.com/2e4765nj

## Introduction

### The Social Challenge of an Aging Population

The demographic changes in mainly industrialized societies include aging of the population because of falling birth rates, increased life expectancy, improved medical care, and adequate nutrition [1,2]. The proportion of people aged 65 years is forecasted to increase from 17% in 2008 to an estimated 30% by 2060, and the number of people aged over 80 years would triple [3]. This age shift is leading to a rapidly growing proportion of the population in need of medical and general care given that the risk of acute and chronic diseases increases with age [4-6]. At the same time, the proportion of healthy caregivers is decreasing [7]. The aging process and the reasons for entry into the need for long-term care are highly individualized, and in Germany, they usually lead to accommodation in nursing homes [8].

Besides losses in physical abilities, cognitive functions in particular are subject to strong age-associated degradation processes in people over 65 years. Population-based epidemiological studies report that 3% to 19% of adults over 65 years of age experience mild cognitive impairment, with more than half of these adults developing dementia within 5 years [9]. Both age-related physical and cognitive impairments lead to increased social isolation in older people in nursing homes [10,11], which in turn has a negative impact on mental health [12]. Empirical research shows that cognitive deterioration processes can be counter-balanced; cognitive training can help healthy older people to significantly improve their cognitive performance and maintain these gains in the long term [13,14].

Different areas of cognitive functioning, such as memory [15], processing speed [16], executive functions [17], and attention [18], can be successfully trained in older people. These findings highlight the relevance and need of prevention, as well as the early detection of cognitive decline [19].

### Opportunities of Prevention

The World Health Organization [20] defines health promotion (prevention) as “[...] a process that enables all people to have a greater degree of self-determination about their health and thus empowers them to strengthen their health.” Numerous studies on demographic change point out that the intensity of prevention and health promotion measures, especially in nursing homes, must increase [21]. Research findings provide a clear indication that health-promoting interventions in long-term inpatient care should not only counteract existing physical and cognitive limitations but also promote remaining skills and health resources [22]. Finally, such interventions would also have positive effects on the number of cases and thus the health care system overall [23].

### Serious Games for Prevention

Serious games are games which, in addition to the fun of the game, have a serious added value. They are suitable for not only imparting knowledge, but also prevention, therapy, and use in care [24]. Although studies have shown that seniors play computer games [25], it is noticeable that there are only a few studies on serious games and older people, with an even more limited number of studies including older adults in care facilities (also limited availability) and even fewer studies on nursing home residents with cognitive impairments [26]. Serious games for seniors are developed with special features. For example, attention must be paid to simple operation, customizable development potential, intuitive and easy-to-remember game mechanics, and game principles [27,28]. Lau et al [29] also emphasized the combination of cognitive challenge and stimulation for physical activity, which makes serious games a multifactorial and extensive prevention medium. Thus, training with serious games provides synergy effects that cannot be achieved with separate training of physical and cognitive skills [30]. Despite the limited research, Chao et al [31] found 22 studies for their meta-analysis that investigated the effects of serious gaming on older people. The results showed promising indications that serious gaming in older people has a positive impact on physical skills, cognition, and psychosocial experience. There are also some studies specifically on serious gaming, cognition, and older people. So far, these studies have yielded diverse results. A very recent meta-analysis [32], which examined 18 randomized studies about the effectiveness of serious gaming on the cognition of older people, reported moderate effects on general cognitive functions and memory. In addition, Mura et al [33] showed in their meta-analysis that people with neurological disabilities benefit from serious gaming in their cognitive functions, especially in executive functions and visuospatial perceptions, but there was no effect on attention and global cognition. These results demonstrate that there are few effects for special cognitive functions, but the effect sizes are often not large enough to be beneficial. Moreover, Sala et al [34] conducted publication-bias analyses in meta-analyses regarding this topic, which suggested that the actual effect of exergames on overall cognitive function is small or even nonexistent.

### Pilot Study on MemoreBox: A Serious Health Game

Launched in 2014, MemoreBox is a science-based, computer-based, gesture-controlled game console, specifically adapted to the needs and abilities of seniors in nursing homes. It aims to promote, activate, and reactivate the seniors’ cognitive, motor, and psychosocial skills in a preventive and therapeutic fashion (RetroBrain R&D, developing company of MemoreBox). In a German pilot study with 72 seniors [35], which was conducted over a period of 6 months, MemoreBox players showed more beneficial development of their cognitive performance, among other health effects, compared with nonplayers (effect of time: playing intervention group, n=28 [*χ*^2^_2_=12.653; *P*=.002; r=2.39]; nonplaying control group, n=29 [*χ*^2^_2_=2.495; *P*=.29]). The positive pilot led to a longitudinal nationwide German study on MemoreBox to evaluate its physical, cognitive, and mental health effects. A detailed methodological discussion of the longitudinal study has been provided elsewhere [36]. This work will evaluate the data from this study relative to cognitive aspects.

The goal of the study is to investigate the impact of the serious game MemoreBox on the cognitive abilities of seniors. Previous considerations with regard to demographic change highlight the need for knowledge about the preventive effect of interventions (physical and psychological). Thus, studies, such as the one presented in this paper, are key to contribute to a growing knowledge base in this area. In order to meet future challenges, it will be essential to turn to novel, resource-oriented, and technology-based ideas that offer possible solutions. One of these ideas could be serious games, and they have the potential to reactivate existing cognitive resources in senior citizens, which is a topic that we aim to explore further in this paper.

## Methods

### Study Design

The current intervention study has a quasiexperimental design with 8 repeated measures. To evaluate the effectiveness of MemoreBox, a large-scale study was conducted in 100 German care facilities, recording and examining a total of 1000 seniors in a playing intervention group and a nonplaying control group over the course of a year. Seniors were allocated to either the intervention group or control group based on their preference, that is, if they wanted to play serious games or just participate in the study without playing.

#### Intervention

MemoreBox (see detailed description above), a serious game that was specifically designed for seniors in care facilities, constituted the focus of the intervention study. MemoreBox was developed a few years ago by health care professionals (physical therapists, medical doctors, and psychologists) based on empirical research. It is a purely computer-based digital intervention that requires the handling of technology, but was designed in a way that it would be as accessible as possible through pure gesture control. Its technical features allow the recording of a player’s movements, including, for instance, inclinations, movement angles, body balance, and movement radius. These movement data can be used in future studies to analyze potential changes in movement or agility. In addition, there is a point system included in the games to promote motivation, and the level of difficulty (sensitivity) can be adjusted.

The intervention included 6 coordinated movement games, which combine preventive, therapeutic, and rehabilitative aspects, aimed at playfully training the physical, mental, and social skills of seniors. Every senior has an individual QR code that records their (movement) data by means of a Kinect camera (Microsoft Corp). The games can be played while sitting or standing, individually or in groups.

**Figure 1:**
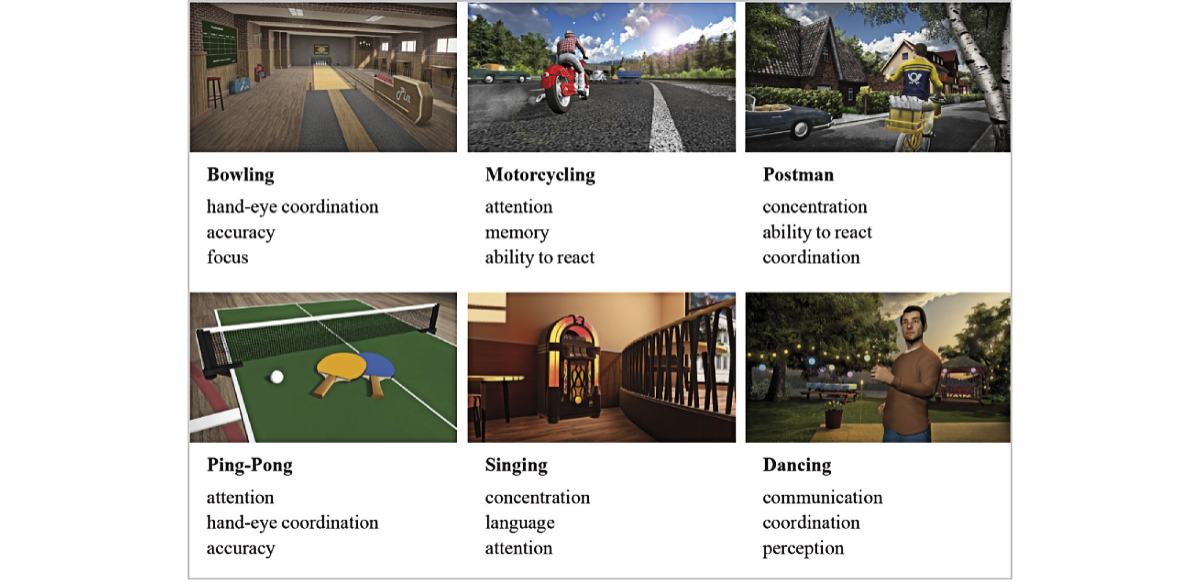
Game modules, and some of their potential therapeutic characters.

The intervention for this study, training through games, was carried out 3 times a week for 1 hour each in a group using a fixed training plan that was developed in advance by therapists. This ensured that each participant used each game (with their different therapeutic foci) once a week. Each training session was supervised by a trained therapist. The preventive training program can be used independently of age-related indications. As shown in Figure 1, there are currently 6 games (motorcycling, bowling, table tennis, singing, postman, and dancing) that are based on everyday activities and focus on different combinations of balance, memory, ability to react, hand-eye coordination, and flexibility.

In addition, MemoreBox provides a record of the amount, duration, and regularity of the game recorded by each person. The data allowed us to differentiate people in the intervention group and subdivide them further into those who regularly played according to the training plan and those who belonged to the intervention group but only exercised irregularly (see the Participants section below).

#### Operationalization

**Figure 2:**
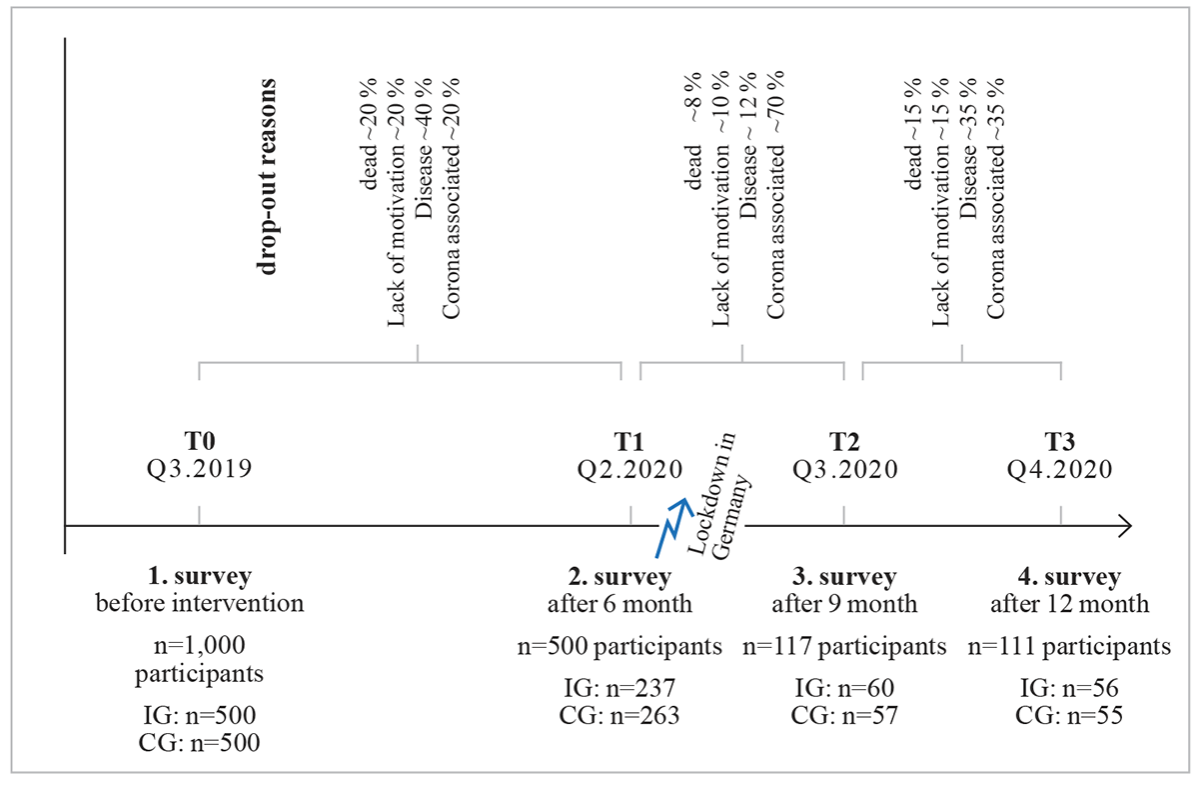
Survey times, periods of the operationalization and drop-out reasons. CG: controls group; IG: intervention group.

Standardized questionnaires on cognition, motor skills, and psychosocial health were administered every 3 to 6 months and were thus completed by participants at 4 to 5 points in time. Given that only cognitive skills are addressed in this study, the corresponding Mini-Mental Status Test (MMST), which has been described in more detail elsewhere [37], was used. It is a screening method for identifying cognitive deficits and monitoring progress. The German-language version was created by Kessler et al [38]. With 30 items, the MMST checks cognitive performance in the following 5 dimensions: temporal and spatial orientation, attention and arithmetic ability, memory, language comprehension, and ability to act. The reliabilities provided in the manual for observer agreement (*r*=0.83) and retest (*r*=0.89; 24 hours apart) are high. There were 4 measurements (Figure 2), where trained nursing staff carried out the MMST. In addition, the objective technical data of the gaming behavior (duration and frequency) were present and were needed.

### Participants

The sample consisted of an intervention group and a control group. The intervention group included 56 participants (38 females and 18 males) aged between 62 and 96 years (mean age 81.84 years, SD 6.78 years). For this study, the technical data from MemoreBox on gaming behavior were used to identify those within the intervention group who played regularly (according to the training plan) in order to obtain more precise insights into the effects of the training. Another intervention group (second intervention group) of 38 senior citizens, who trained regularly for 1 year, was identified. Of the 38 participants in the second intervention group (regular end of the game), 28 (74%) were females and 10 (26%) were males, with an age range of 71 to 96 years (mean 83.61 years, SD 5.38 years). The control group included 55 participants (41 females and 14 males) aged between 60 and 107 years (mean age 84.24 years, SD 9.33 years). The distributions of age (mean 83.03 years, SD 8.2 years) and gender (66 females, 71%; 28 males, 29%) of the total sample roughly corresponded to the current findings on the need for care in Germany [39].

All participants were residents of care facilities in Germany. A total of 100 care facilities were included. When selecting the facilities, care was taken to ensure that the facility structure was very diverse (eg, private, state, church, city, and country). For both groups, severe mental or neurological illnesses and age below 60 years were exclusion criteria. In addition, the health status, comorbidities, and medications in both groups were surveyed.

#### Recruitment

After the nursing staff of the facilities passed on the information materials to the residents, the seniors could volunteer to participate in the study. A similar procedure was used to assign participants to the intervention or control groups. The participants voluntarily assigned themselves to 1 of the 2 groups. Given that this voluntary group assignment led to unequal groups with different parameters, the group data were parallelized for comparison purposes after data collection was completed.

#### Dropout and Missing Data Resulting From the COVID-19 Pandemic

The global COVID-19 pandemic, which has caused major changes in care facilities in Germany at least since March 2020, led to the inevitable interruption of the study and thus of the planned surveys in most of the participating care facilities. Due to the large number of nationally participating institutions, the individual on-site situation and the local restrictions imposed because of COVID-19 differed greatly from one care facility to the next. In 11 nursing homes, the study design could be continued unhindered based on the training plan because of special facility conditions, which ultimately led to a sample size of 111 (56 in the intervention group and 55 in the control group) in this study (Figure 2). The surveys were subject to a very brief interruption (3 months), which led to an analyzable and sufficiently interpretable data set in the sense of the originally intended study design for 4 measurement times with different intervals. Additionally, the intervention group (n=56) was further divided into the following 2 groups: a group of participants for whom the data in MemoreBox indicated that they had participated in the training relatively regularly despite the pandemic conditions (second intervention group, n=38) and a group of participants for whom the data showed that they had participated irregularly (n=18).

The MMST was originally planned to be administered every 6 months. However, due to the changes in the nursing homes as a result of COVID-19, an additional measurement was carried out after the first easing of the lockdown in the summer of 2020, to have a kind of “second start value.” This step resulted in 4 MMST measurement times, with the interval between the first 2 measurements being 6 months and the further 2 intervals being 3 months each (Figure 2).

### Statistical Analysis

To ensure comparability, the intervention and control groups were compared and parallelized in terms of the characteristics of the dependent variables at baseline (T0). For the statistical significance test, the confidence interval was set at a level of α=.05. To examine the 2 intervention groups in comparison with the control group, a mixed analysis of variance (ANOVA) was used. The dependent variable was cognitive impairment, the time factor with 4 values (T0 [Q3 2019], T1 [Q2 2020], T2 [Q3 2020], and T3 [Q4 2020]) functioned as an inner-subject factor, and group membership functioned as a between-subject factor (2 factor levels).

### Ethics Approval

This study was approved by the Ethics Committee of Charite Berlin (Ethikausschuss am Campus Benjamin Franklin; review number: EA4/035/19).

## Results

### Overview

We outline the results and separate the intervention group into the original intervention group and the second intervention group (ie, those seniors who completed the training for a year according to the plan).

### Baseline Comparison/Descriptive Statistics

There were no noteworthy differences between the demographic and main variables in the overall sample. Moreover, in the newly created classification, the groups at time T0 (baseline) did not differ significantly in demographic and main variables (Tables 1 and 2).

**Table 1:** Baseline measurement of variables and their differences between the intervention group and control group.

Variable	Intervention group (N=56), mean (SD)	Control group (N=55), mean (SD)	*t* (*df*)	*P* value	95% CI
Age	81.84 (6.78)	84.24 (9.33)	−1.55 (98.58)	.13	−0.67 to 0.08
Care level^a^	2.49 (0.89)	2.67 (0.83)	−1.02 (99)	>.99	−0.60 to 0.19
Financial medium score^b^	2.03 (0.88)	2.00 (1.04)	0.121 (68)	.90	−0.44 to 0.50
Health condition score^c^	3.28 (1.07)	3.10 (1.27)	0.79 (103)	.43	−0.23 to 0.54
Health behavior score^d^	2.34 (0.79)	2.53 (0.97)	−1.11 (109)	.27	−0.58 to 0.16
Health assessment score^e^	2.84 (0.80)	2.89 (0.83)	−0.33 (109)	.74	−0.44 to 0.31
MMST^f^ mean score	1.32 (0.25)	1.29 (0.26)	0.536 (109)	.59	−0.27 to 0.47
MMST total score	24.77 (4.60)	24.47 (4.86)	0.329 (109)	.74	−0.31 to 0.43

^a^0 (no need for care) to 5 (most severe impairments).

^b^0=<€1000; 1=€1000-€1500; 2=€1500-€2000; >€2000.

^c^0 (healthy) to 5 (chronically ill).

^d^0 (not taking care of their health) to 5 (taking great care of their health).

^e^0 (“I rate my health as very bad”) to 5 (“I rate my health as very good”).

^f^MMST: Mini-Mental Status Test.

**Table 2:** Baseline measurement of variables and their differences between the second intervention group (regular players) and control group.

Variable	Second intervention group (N=38), mean (SD)	Control group (N=55), mean (SD)	*t* (*df*)	*P* value	95% CI
Age	83.61 (5.38)	84.24 (9.33)	−0.412 (88.54)	.68	−0.49 to 0.34
Care level^a^	2.42 (0.94)	2.67 (0.83)	−1.30 (82)	.20	−0.72 to 0.15
Financial medium score^b^	2.00 (0.83)	2.00 (1.04)	0.00 (58)	>.99	−0.51 to −0.51
Health condition score^c^	3.41 (1.04)	3.10 (1.27)	1.22 (86)	.23	−0.16 to 0.68
Health behavior score^d^	2.37 (0.75)	2.53 (0.97)	−0.84 (91)	.40	−0.59 to 0.24
Health assessment score^e^	2.89 (0.73)	2.89 (0.83)	0.02 (91)	.98	−0.41 to 0.42
MMST^f^ mean score	1.32 (0.23)	1.29 (0.26)	0.60 (91)	.55	−0.29 to 0.54
MMST total score	24.76 (4.25)	24.47 (4.86)	0.30 (91)	.77	−0.35 to 0.48

^a^0 (no need for care) to 5 (most severe impairments).

^b^0=<€1000; 1=€1000-€1500; 2=€1500-€2000; >€2000.

^c^0 (healthy) to 5 (chronically ill).

^d^0 (not taking care of their health) to 5 (taking great care of their health).

^e^0 (“I rate my health as very bad”) to 5 (“I rate my health as very good”).

^f^MMST: Mini-Mental Status Test.

### Outcome

There was no normal distribution in the sample, which can be neglected with a sample size of >30 [8]. In addition, the *F* tests carried out showed similar results. The sphericity was also not given (0.04). Owing to the violation of this requirement, a Greenhouse-Geisser correction of the degrees of freedom was carried out.

**Figure 3:**
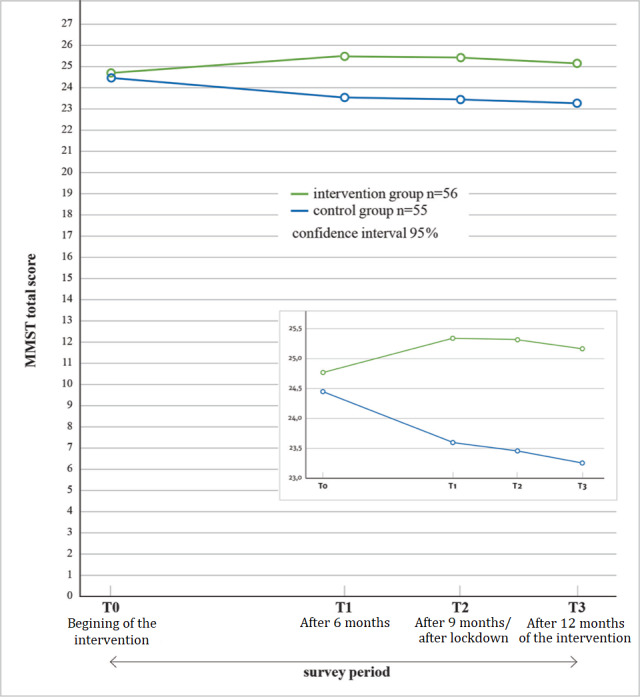
Results of the Mini-Mental Status Test (MMST) of the intervention group (all participants) and control group over time.

First, we analyzed the whole sample (N=111) to explore if there was a potential distinction between the intervention group and the control group. The mixed ANOVA showed no statistically significant interaction between time and group membership (*F*_2.710,295.379_=1.942; *P*<.13; partial *η*²=0.018). There was also no significant main effect for time, which corresponded to no significant difference over time (*F*_2.710,295.379_=0.383; *P*=.75; partial *η*²=0.04; Figure 3). There was also no significant main effect for group membership (*F*_1.109_=2.405; *P*=.12; partial *η*²=0.022).

The clear tendencies of the MMST estimates of both participant groups, which are clearly shown in Figure 3 but were not found to be statistically significant, were another reason for a closer look at the intervention group and the decision to further divide participants based on the available MemoreBox data, that is, creating the second intervention group (n=38) based on the amount, duration, and regularity of game play.

**Figure 4:**
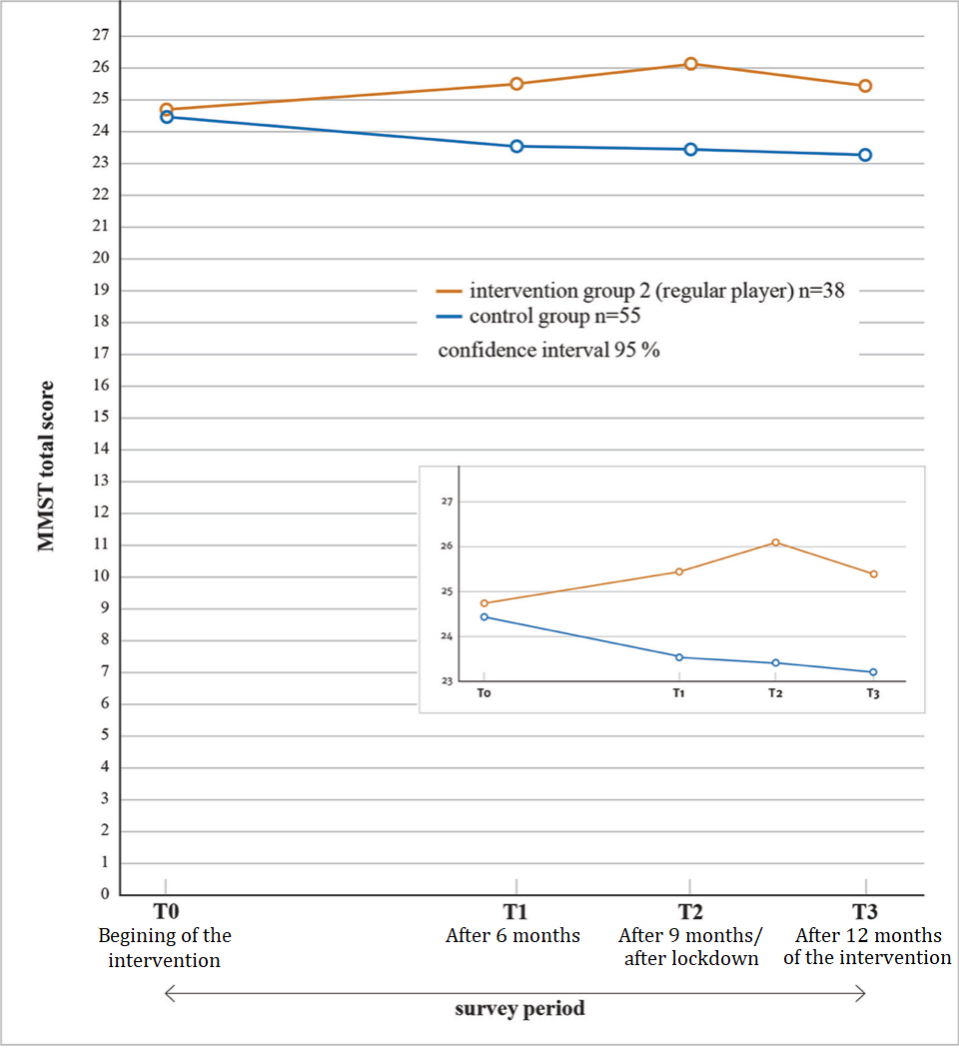
Results of the Mini-Mental Status Test (MMST) of the second intervention group playing regularly and the control group at all 4 measurement times.

On performing mixed ANOVA for group differences between the second intervention group and the control group, no statistically significant interaction between time and group membership was found (*F*_3.273_=2.574; *P*<.054; partial *η*²=0.028; Figure 4).

However, the level of significance was only just exceeded, and at a level of significance of 10%, a clear main effect and thus an interaction of time and group affiliation could be identified. Hence, we could conclude that there were clear tendencies that playing with MemoreBox over a year improved the cognitive abilities of the participants who played regularly, whereas they deteriorated in the control group both in real numbers and in statistical comparison. The main effect of time (*F*_3.273_=0.337; *P*=.78; partial *η*²=0.004) and group membership (*F*_1.91_=2.701; *P*=.10; partial *η*²=0.029) for this ANOVA did not show statistical significance. The subsequent *t* tests (Table 3) showed a statistically significant difference between the groups in terms of their MMST values after 9 months of the intervention (t_88.1_=−2.394; *P*=.02). This effect was already apparent after 6 months, but was easily canceled out after 12 months.

**Table 3:** Results of the *t* test for the intervention and control groups at 4 different measurement times.

Time point comparison^a^	Control group			Second intervention group			Control group vs second intervention group		
	*t* (*df*)	*P* value	*t* (*df*)		*P* value	Time point		*t* (*df*)	*P* value
T0–T1	1.433 (54)	.16	−1.341 (37)		.19	T0		−0.298 (91)	.77
T1–T2	0.379 (54)	.71	1.156 (37)		.26	T1		1.725 (89.7)	.09
T2–T3	−0.221 (54)	.83	1.357 (37)		.18	T2		2.349 (88.1)	.02^b^
T0–T3	1.681 (54)	.10	−0.893 (37)		.38	T3		1.597 (91)	.11

^a^T0: Q3 2019, T1: Q2 2020, T2: Q3 2020, T3: Q4 2020.

^b^Significant at 95% CI; N=93.

## Discussion

### Principal Findings

The baseline comparison showed that the intervention and control groups did not differ statistically in any variable at the beginning of the intervention and were therefore deemed comparable. The relevant sociodemographic variables roughly corresponded to the current findings about the need for long-term care in Germany [39].

The mixed ANOVA showed no significant impact on the cognitive abilities of those subjects who played regularly over the course of a year compared with the control group. However, owing to the size of the sample and the statistical significance, we hypothesize that the intervention had a potential impact on cognition.

This impact could not be detected among irregular players. An independent samples *t* test between the groups after 9 months of the intervention showed a statistically significant difference between the groups, which was not visible at earlier measurement times.

Furthermore, the figures and graphics indicate development tendencies in a way that the control group estimates decrease over the period of the investigation (1 year) from an initial average MMST value of 24.47 to an average MMST value of 23.25. However, this change was not statistically significant. Nevertheless, this development was understandable, given that a decline in cognitive abilities can be expected for this group of participants (ie, senior citizens) within 1 year [40-42]. In addition, within a year, the MMST values for the total sample fell below the frequently set limit value for normal cognitive function (24 points), which may indicate normal dementia development. However, by contrast, the values of the second intervention group showed an opposite trend (from 24.76 to 25.42).

Overall, despite the described limitations, the results clearly showed a tendency that supports the effectiveness of the intervention and suggests a positive impact on the cognitive abilities of seniors in nursing homes. Thus, the results of this study contribute considerably to our knowledge base in this novel and still underresearched area by providing insights into the challenges and complexities, as well as potential developments and implementations of serious games that can be further explored in future research. Moreover, the results indicate that serious games (ie, here MemoreBox) can have a positive impact on the cognitive abilities of seniors and should therefore be increasingly recognized and implemented to provide opportunities for engaging health promotion.

### Limitations

The quasiexperimental design, the innovative character of the project, and the interruption by the global pandemic caused limitations. The study design involved nonrandomized assignment, no given double-blind procedure, and a small sample size.

To conduct a survey study over the long period of 12 months and avoid excessive drop-out rates, it is particularly helpful for research projects in the geriatric field to keep the motivation to participate as high as possible by means of voluntary instead of randomized allocation. The baseline comparison should represent the greatest possible compensation for this limitation.

Another major limitation is the small size of the sample that was analyzed at the end. The attrition from the initial 1000 participants to 111 usable data sets (approximately 10%) was very large. Due to the special circumstances of the participants (age, illnesses, care, and morbidity) and the high effort for the intervention group (1 year, 3 times a week commitment), the target group had a high drop-out rate from the start, which can be expected [43]. The sample size may also be a reason for the lack of statistically significant differences between the groups. A corresponding post-hoc power analysis with an alpha level of 5% and a target power of 80% (90%) showed a minimum number of 179 (231) cases to show a significant interaction effect with moderate effect sizes (*η*Ç=0.25) with a mixed ANOVA in 2 groups and 4 measurements.

### Comparison With Prior Work

Regarding the cognitive ability of the study participants, the results showed that the condition in the control group deteriorated slightly within 1 year, a finding that is in line with previous research [40-42]. However, it can be assumed that regular players at least maintained their cognitive abilities on average and that the progression of cognitive impairments can be slowed down by training. These effects and tendencies confirmed the findings from a previous pilot study by Trauzettel [35], who also tested the influence of MemoreBox on cognitive abilities in 2 nursing homes and found a significant improvement in the MMST values over a 6-month survey period, as well as a significant difference between the intervention group and control group at the last measurement time. The fact that therapeutic computer-based training had a positive effect on cognitive performance shows consistency with other research findings [44]. This is particularly important regarding the age-related decline in cognitive abilities [40]. Additionally, it means that success can lie not only in growth, but also in maintaining cognitive abilities [45]. Moreover, the results of this study coincide with other studies in which comparable interventions with therapeutic game consoles brought about multidimensional improvements in older people [46-49]. In addition, reviews reflect the high potential of serious games as an efficient and motivating component in prevention and health promotion [28,29,50-52].

Apart from the already reported contribution of the study to advancing the knowledge base in this new research area and the clear influence of the intervention in older people, it is also important to highlight the “nursing home” as a novel space and focus in research. Given that nursing homes require a considerable amount of social, financial, and health resources, this study focused on the implementation of serious games as a means of potentially aiding with constrains in these resources. It focused on the efficacies and points to positive effects relative to the 3 resources. Thus, our study clearly highlights the importance of this type of research as well as the attention this area of research should receive in the future.

In the following text, it will also be critically discussed why the results presented here do not show statistical significance, as the literature suggests. The initial values of all participants were in a high range of approximately 24 points, which is not considered to be dangerous. These high baseline values may be a reason for the ambiguous significance in the sense of an improvement, since older people with very low cognitive baseline values tend to benefit more from cognitive interventions than people who already show higher values before training [53]. It remains to be seen whether the second intervention group can maintain the generally stable MMST values and whether this would be reflected in a comparison with the control group over a significantly longer intervention period.

Another reason can be personal influences and associated other everyday activities. As previous research shows, cognitive activity throughout the life of a person is seen as an important factor influencing cognitive performance in old age [54,55]. This could mean that the influence of the intervention is less than assumed, since the level of activity in the previous life of the individual played a more important role. Since improvements in cognitive performance are associated, among other things, with increased health-related quality of life [56] and, on the contrary, a cognitive decline is associated with a lower health-related quality of life [57], there might have been other factors that impacted the results. The Hawthorne effect [58] could be one factor, that is, positive effects on the examined variables solely through conscious participation in the study can (unconsciously) motivate the control group to change their behavior. This could have resulted in stabilizing, compensatory, or diffusing effects that do not correctly reflect the characteristics in the constructs. Possible consequences are a lack of reliability of the implemented intervention and a limited construct validity, which could have resulted in an approximation of the measured values for the second intervention group and control group [59].

At the same time, the game concept of MemoreBox is based on stimulating interactivity and communication with other participants. Epidemiological studies have shown that social relationships are highly relevant to cognitive health and can even reduce the risk of death [60]. Being with other people involves cognitive stimulation through verbal and nonverbal communication. A lack of social relationships, on the other hand, can favor dementia [61]. The changing group size from 5 persons initially to, in some cases, only 1 person at the end of the 1-year intervention could therefore also have had an impact on the results.

### Future Perspective

Follow-up studies are much needed to evaluate the continued effects of the changes and investigate the effectiveness beyond the current influence of the game. Future studies should take into account a new group, which receives a different multimodal offer (as similar as possible, but, for example, guided by therapists), in order to differentiate more precisely the specific areas of impact of the given serious game. Additionally, analyzing the exact impact on preventive and health-promoting changes could also be the focus of future research, which would contribute further to the literature that has outlined the relatively high benefits of serious games.

Establishing a high scientific standard requires not only a second intervention group, but also various more specialized instruments to document motor movements, which, in turn, can potentially provide insights into the effectiveness of serious games for different motor skill sets.

Furthermore, it is essential to carry out studies that have a larger sample size. Owing to the already mentioned challenges of recruitment and stability of the target group, there is a global lack of studies with a high number of participants. Future studies, like the present one, could obtain a larger sample size by establishing interventions directly in nursing homes.

In addition to focusing on a higher number of participants, future studies should also concentrate on follow-up examinations to document long-term effects and, in general, collect data over a fundamentally longer period of time. The potential reactivation/preventive effects relative to certain motor skills seem to take a long time, especially for the target group, as shown by the results reported in this paper, as well as in other relevant literature. Therefore, the duration of the studies must be adjusted in order to better assess the actual long-term benefits of serious games for the motor skills of seniors.

### Conclusions

While confirming the current state of the research field, the results of this study showed that the intervention had an impact on the cognitive abilities of seniors, provided that they regularly played the serious game of MemoreBox. A particularly novel aspect of this study is that it was carried out in the actual care sector. The authors see the study as a continuation of a pilot I study [35]. Both studies have made it their task to consider the behaviors and circumstances of senior citizens in a resource-oriented and setting-related manner and thus to make a scientific contribution to the limited research in this area [35]. The contribution of the paper is that it shows the positive influence of serious games on the cognitive abilities of older people and can thus be seen as an important building block toward better understanding of preventive effects.

Implementing an easy-to-use serious game as an effective (prevention) tool and making it part of the standard care in nursing homes might contribute considerably to the weak health care system, in which there tends to be a lack of activating offers for senior citizens in partially inpatient care facilities [62].

## Abbreviations

ANOVA: analysis of variance
MMST: Mini-Mental Status Test

## References

[1] Felder S (2012). [Health care expenditures and the aging population]. Bundesgesundheitsblatt Gesundheitsforschung Gesundheitsschutz.

[2] Brown GC (2015). Living too long: the current focus of medical research on increasing the quantity, rather than the quality, of life is damaging our health and harming the economy. EMBO Rep.

[3] (2008). Ageing characterises the demographic perspectives of the European societies - Issue number 72/2008. European Commission.

[4] Fisk A, Rogers W (1997). Handbook of Human Factors and the Older Adult.

[5] Jaul E, Barron J (2017). Age-Related Diseases and Clinical and Public Health Implications for the 85 Years Old and Over Population. Front Public Health.

[6] Jacobs K, Kuhlmey A, Greß S, Klauber J, Schwinger A (2017). Pflege-Report 2019 Mehr Personal in der Langzeitpflege - aber woher?.

[7] Garms-Homolová V, Kuhlmey A, Schaeffer D (2008). Prävention bei Hochbetagten. Alter, Gesundheit und Krankheit.

[8] Bortz J, Schuster C (2010). Statistik für Human- und Sozialwissenschaftler.

[9] Gauthier S, Reisberg B, Zaudig M, Petersen RC, Ritchie K, Broich K, Belleville S, Brodaty H, Bennett D, Chertkow H, Cummings JL, de Leon M, Feldman H, Ganguli M, Hampel H, Scheltens P, Tierney MC, Whitehouse P, Winblad B (2006). Mild cognitive impairment. The Lancet.

[10] Hämmig O (2019). Correction: Health risks associated with social isolation in general and in young, middle and old age. PLoS One.

[11] Nicholson NR (2012). A review of social isolation: an important but underassessed condition in older adults. J Prim Prev.

[12] Landeiro F, Barrows P, Nuttall Musson E, Gray AM, Leal J (2017). Reducing social isolation and loneliness in older people: a systematic review protocol. BMJ Open.

[13] Neely AS, Backman L (2006). Maintenance of gains following multifactorial and unifactorial memory training in late adulthood. Educational Gerontology.

[14] Schaie KW (1997). Intellectual Development in Adulthood: The Seattle Longitudinal Study.

[15] Smith GE, Housen P, Yaffe K, Ruff R, Kennison RF, Mahncke HW, Zelinski EM (2009). A cognitive training program based on principles of brain plasticity: results from the Improvement in Memory with Plasticity-based Adaptive Cognitive Training (IMPACT) study. J Am Geriatr Soc.

[16] Edwards JD, Wadley VG, Vance DE, Wood K, Roenker DL, Ball KK (2005). The impact of speed of processing training on cognitive and everyday performance. Aging Ment Health.

[17] Uchida S, Kawashima R (2008). Reading and solving arithmetic problems improves cognitive functions of normal aged people: a randomized controlled study. Age (Dordr).

[18] Mozolic JL, Long AB, Morgan AR, Rawley-Payne M, Laurienti PJ (2011). A cognitive training intervention improves modality-specific attention in a randomized controlled trial of healthy older adults. Neurobiol Aging.

[19] Kolassa I, Glöckner F, Leirer V, Diener C (2010). Neuronale Plastizität bei gesundem und pathologischem Altern. Altern gestalten - Medizin, Technik, Umwelt.

[20] World Health Organization (1986). Ottawa charter for health promotion. Health Promot Int.

[21] Lippke S, Kuhlmann T (2013). Gesundheitsförderungsmaßnahmen für ältere Menschen mittels neuer Medien. Zeitschrift für Gesundheitspsychologie.

[22] Schaeffer D, Büscher A (2009). [Options for health care promotion in long-term care: empirical evidence and conceptual approaches]. Z Gerontol Geriatr.

[23] Brookmeyer R, Gray S, Kawas C (1998). Projections of Alzheimer's disease in the United States and the public health impact of delaying disease onset. Am J Public Health.

[24] Nguyen T, Ishmatova D, Tapanainen T, Liukkonen T, Katajapuu N, Makila T, Luimula M (2017). Impact of Serious Games on Health and Well-being of Elderly: A Systematic Review. Proceedings of the 50th Hawaii International Conference on System Sciences.

[25] (2020). Jahresreport der deutschen Games-Branche. Game.

[26] Saint-Maurice Pedro F, Troiano RP, Matthews CE, Kraus WE (2018). Moderate-to-Vigorous Physical Activity and All-Cause Mortality: Do Bouts Matter?. J Am Heart Assoc.

[27] Wiemeyer J (2018). Spielerische Förderung körperlicher Aktivität von Älteren. Präv Gesundheitsf.

[28] Wiemeyer J, Kliem A (2011). Serious games in prevention and rehabilitation—a new panacea for elderly people?. Eur Rev Aging Phys Act.

[29] Lau HM, Smit JH, Fleming TM, Riper H (2016). Serious Games for Mental Health: Are They Accessible, Feasible, and Effective? A Systematic Review and Meta-analysis. Front Psychiatry.

[30] Smeddinck JD, Gerling KM, Malaka R (2014). Anpassbare Computerspiele für Senioren. Informatik Spektrum.

[31] Chao Y, Scherer YK, Montgomery CA (2015). Effects of using Nintendo Wii™ exergames in older adults: a review of the literature. J Aging Health.

[32] Yen H, Chiu H (2021). Virtual Reality Exergames for Improving Older Adults' Cognition and Depression: A Systematic Review and Meta-Analysis of Randomized Control Trials. J Am Med Dir Assoc.

[33] Mura G, Carta MG, Sancassiani F, Machado S, Prosperini L (2018). Active exergames to improve cognitive functioning in neurological disabilities: a systematic review and meta-analysis. Eur J Phys Rehabil Med.

[34] Sala G, Tatlidil KS, Gobet F (2021). Still no evidence that exergames improve cognitive ability: A commentary on Stanmore et al. (2017). Neurosci Biobehav Rev.

[35] Trauzettel F Evaluation präventiver und gesundheitsförderlicher Aspekte von Serious Games im Alter. Humboldt-Universität zu Berlin.

[36] Kleschnitzki J, Großmann I, Arndt S, Beyer R, Beyer L (2020). Analysis of a study design which evaluates a serious game for cognitive and motor activation of senior citizens. Empirische Evaluationsmethoden.

[37] Folstein MF, Folstein SE, McHugh PR (1975). “Mini-mental state”. Journal of Psychiatric Research.

[38] Kessler J, Denzler PE, Markowitsch HJ (1990). Mini-Mental-Status Test (MMST). Deutsche Fassung.

[39] Matzk S, Tsiasioti C, Behrendt S, Jürchott K, Schwinger A, Jacobs K, Kuhlmey A, Greß S, Klauber J, Schwinger A (2020). Pflegebedürftigkeit in Deutschland. Pflege-Report 2020.

[40] Park DC, Smith AD, Lautenschlager G, Earles JL, Frieske D, Zwahr M, Gaines CL (1996). Mediators of long-term memory performance across the life span. Psychol Aging.

[41] Oswald W, Gatterer G, Fleischmann U (2008). Gerontopsychologie Grundlagen und klinische Aspekte zur Psychologie des Alterns.

[42] Dechamps A, Diolez P, Thiaudière E, Tulon A, Onifade C, Vuong T, Helmer C, Bourdel-Marchasson I (2010). Effects of exercise programs to prevent decline in health-related quality of life in highly deconditioned institutionalized elderly persons: a randomized controlled trial. Arch Intern Med.

[43] Chang CH, Yang H, Tang G, Ganguli M (2009). Minimizing attrition bias: a longitudinal study of depressive symptoms in an elderly cohort. Int Psychogeriatr.

[44] Schmiedek F, Lövdén M, Lindenberger U (2010). Hundred Days of Cognitive Training Enhance Broad Cognitive Abilities in Adulthood: Findings from the COGITO Study. Front Aging Neurosci.

[45] Krupp LB, Charvet LE (2019). Long-term Cognitive Consequences for Patients With Pediatric-Onset Multiple Sclerosis. JAMA Neurol.

[46] Aarhus R, Grönvall E, Larsen S, Wollsen S (2011). Turning training into play: Embodied gaming, seniors, physical training and motivation. Gerontechnology.

[47] Anguera JA, Boccanfuso J, Rintoul JL, Al-Hashimi O, Faraji F, Janowich J, Kong E, Larraburo Y, Rolle C, Johnston E, Gazzaley A (2013). Video game training enhances cognitive control in older adults. Nature.

[48] Miller KJ, Dye RV, Kim J, Jennings JL, O'Toole E, Wong J, Siddarth P (2013). Effect of a computerized brain exercise program on cognitive performance in older adults. Am J Geriatr Psychiatry.

[49] Rand D, Kizony R, Weiss P (2004). Virtual reality rehabilitation for all: Vivid GX versus Sony PlayStation II EyeToy. Proceedings of the 5th international conference on disability, virtual reality and associated technologies.

[50] Fleming TM, Cheek C, Merry SN, Thabrew H, Bridgman H, Stasiak K, Shepherd M, Perry Y, Hetrick S (2015). Juegos serios para el tratamiento o la prevención de la depresión: una revisión sistemática. RPPC.

[51] Smeddinck J, Siegel S, Herrlich M (2013). Adaptive difficulty in exergames for Parkinson's disease patients. GI '13: Proceedings of Graphics Interface 2013.

[52] Wiemeyer J (2008). Multimedia in sport – between illusion and realism. WIT Press.

[53] Roheger M, Kessler J, Kalbe E (2019). Structured Cognitive Training Yields Best Results in Healthy Older Adults, and Their ApoE4 State and Baseline Cognitive Level Predict Training Benefits. Cogn Behav Neurol.

[54] Hertzog C, Kramer AF, Wilson RS, Lindenberger U (2008). Enrichment Effects on Adult Cognitive Development: Can the Functional Capacity of Older Adults Be Preserved and Enhanced?. Psychol Sci Public Interest.

[55] Lövdén M, Ghisletta P, Lindenberger U (2005). Social participation attenuates decline in perceptual speed in old and very old age. Psychol Aging.

[56] Cohen RA, Moser DJ, Clark MM, Aloia MS, Cargill BR, Stefanik S, Albrecht A, Tilkemeier P, Forman DE (1999). Neurocognitive functioning and improvement in quality of life following participation in cardiac rehabilitation. Am J Cardiol.

[57] Carmelli D, Swan GE, LaRue A, Eslinger PJ (1997). Correlates of change in cognitive function in survivors from the Western Collaborative Group Study. Neuroepidemiology.

[58] Roethlisberger FJ, Dickson WJ, Wright HA (1993). Management and the Worker: An Account of a Research Program Conducted by the Western Electric Company, Hawthorne Works, Chicago.

[59] Shadish WR, Cook TD, Campbell DT (2002). Experimental and Quasi-Experimental Designs for Generalized Causal Inference.

[60] Zunzunegui M, Alvarado BE, Del Ser T, Otero A (2003). Social networks, social integration, and social engagement determine cognitive decline in community-dwelling Spanish older adults. J Gerontol B Psychol Sci Soc Sci.

[61] Ballesteros S, Kraft E, Santana S, Tziraki C (2015). Maintaining older brain functionality: A targeted review. Neurosci Biobehav Rev.

[62] Blüher S, Kuhlmey A, Richter M, Hurrelmann K (2016). Demographischer Wandel, Altern und Gesundheit. Soziologie von Gesundheit und Krankheit.

